# Clinical Pharmacology of Furosemide in Neonates: A Review

**DOI:** 10.3390/ph6091094

**Published:** 2013-09-05

**Authors:** Gian Maria Pacifici

**Affiliations:** Section of Pharmacology, Department of Translational Research and New Technologies in Medicine and Surgery, University of Pisa, Pisa 56100, Italy; E-Mail: pacifici@biomed.unipi.it; Tel: +39-050-2218-721; Fax: +39-050-2218-717.

**Keywords:** furosemide, neonate, metabolism, pharmacokinetics, pharmacodynamics, continuous infusion, extracorporeal membrane oxygenation, side-effects

## Abstract

Furosemide is the diuretic most used in newborn infants. It blocks the Na^+^-K^+^-2Cl^−^ symporter in the thick ascending limb of the loop of Henle increasing urinary excretion of Na^+^ and Cl^−^. This article aimed to review the published data on the clinical pharmacology of furosemide in neonates to provide a critical, comprehensive, authoritative and, updated survey on the metabolism, pharmacokinetics, pharmacodynamics and side-effects of furosemide in neonates. The bibliographic search was performed using PubMed and EMBASE databases as search engines; January 2013 was the cutoff point. Furosemide half-life (t_1/2_) is 6 to 20-fold longer, clearance (Cl) is 1.2 to 14-fold smaller and volume of distribution (Vd) is 1.3 to 6-fold larger than the adult values. t_1/2_ shortens and Cl increases as the neonatal maturation proceeds. Continuous intravenous infusion of furosemide yields more controlled diuresis than the intermittent intravenous infusion. Furosemide may be administered by inhalation to infants with chronic lung disease to improve pulmonary mechanics. Furosemide stimulates prostaglandin E2 synthesis, a potent dilator of the patent ductus arteriosus, and the administration of furosemide to any preterm infants should be carefully weighed against the risk of precipitation of a symptomatic patent ductus arteriosus. Infants with low birthweight treated with chronic furosemide are at risk for the development of intra-renal calcifications.

## 1. Introduction

Diuretics increase the rate of urine flow and Na^+^ excretion and are used to adjust the volume and/or the composition of body fluids. The basic urine-forming unit of the kidney is the nephron, which consists of a filtering apparatus, the glomerulus, connected to a long tubular portion that reabsorbs and conditions the glomerular ultrafiltration. Furosemide increases the delivery of solutes out of the loop of Henle, is a sulphonamide derivative, and is the most commonly used diuretic in the newborn period [1,2]. Given in excessive amounts, furosemide can lead to dehydration and electrolytic depletion [3].

Around the year 1960 “loop” diuretics (furosemide, ethacrynic acid and bumetanide) were developed. These block the Na^+^-K^+^-2Cl^−^ cotransport system in the ascending limb [4] and inhibit Na^+^, Cl^−^ and K^+^ entering the tubular cell. Loop diuretics are highly efficacious, and for this reason, are called “high-ceiling-diuretics”.

The flux of Na^+^, K^+^ and Cl^−^ from the lumen into the epithelial cells in the thick ascending limb is mediated by a Na^+^-K^+^-2Cl^−^ symporter. This symporter captures free energy in the Na^+^ electrochemical gradient established by the basolateral Na^+^ pump and provides for “uphill” transport of K^+^ and Cl^−^ into the cell. Abolition of the transepithelial potential difference also results in a marked increase in Ca^2+^ and Mg^2+^ excretion, with consequent increase of urine pH.

Furosemide has weak carbonic anhydrase-inhibiting activity. Drugs with carbonic anhydrase-inhibiting activity increase urinary excretion of HCO_3_^−^ and phosphate. By blocking active NaCl reabsorption in the thick ascending limb, furosemide interferes with a critical step in the mechanism that produces a hypertonic medullary interstitium. Therefore, furosemide blocks the kidney’s ability to concentrate urine during hydropenia [3].

Furosemide binds extensively to plasma proteins and bilirubin displacement is negligible when using normal doses of furosemide. Delivery of this drug to the tubules by filtration is limited and it enters the tubules by tubular secretion. In adults, average bioavailability of furosemide is 71 ± 35% and it ranges from 43% to 73% [5]. In neonates, mean bioavailability is 84.3% (range 56% to 106%) [6]. In infants, time to peak effect when given intravenously is 1 to 3 h [7]. There is a remarkable interindividual variability in the kinetic parameters of furosemide in neonates. The half-life (t_1/2_) is 6 to 20-fold longer, clearance (Cl) is 1.2 to 14-fold smaller and volume of distribution (Vd) is 1.3 to 6-fold larger than the adult values.

In neonates, duration of the effect is approximately 6 h, although half-life (t_1/2_) may be as long as 67 h in the most immature newborn infants [7]. Furosemide may be administered as intravenous continuous infusion and yields more controlled diuresis as compared with intermittent intravenous infusion. Extra corporeal membrane oxygenation (ECMO) is a potentially life-saving therapy for neonates suffering from severe respiratory failure. The most common diagnosis for which ECMO is performed are diaphragmatic hernia, meconium aspiration syndrome, and pneumonia [8]. The variable renal function, and the altered furosemide pharmacokinetics, that range from 0.02 to 0.17 mg/kg/h [9], make the dosing schedule of furosemide for ECMO largely empirical. Furosemide may be administered by inhalation to preterm infants with chronic lung disease [10] to improve pulmonary mechanics [11].

Furosemide stimulates the renal synthesis of prostaglandin E2 [12], a potent dilator of the patent ductus arteriosus, and the administration of furosemide to any patient with respiratory distress syndrome should be carefully weighed against the risk of precipitation of a symptomatic patent ductus arteriosus [11,13].

Furosemide can cause ototoxicity that manifests as tinnitus, hearing impairment, deafness, vertigo, and a sense of fullness in the ears [3]. Hearing impairment and deafness are usually, but not always, reversible. Ototoxicity occurs most frequently with rapid intravenous administration and least frequently with oral administration [3]. Heidland and Wigand [14] suggested that furosemide should be given at a rate less than 4 mg/min to adult patients to avoid hearing loss. In infants, furosemide blood levels should not exceed 50 µg/mL [15]. The chronic treatment of furosemide to low birthweight infants may develop intra-renal calcification [16]. The vulnerability of extreme immaturity and the underdevelopment of renal function may be the most important variables. Hypercalciuria is common in very low birthweight infants, yet not all develop nephrocalcinosis.

The work on the clinical pharmacology of furosemide in neonates was published in different journals during the period 1970 to 2013 and the relative information is scattered. The present article aims to gather together and to review the studies on the metabolism, pharmacokinetics, pharmacodynamics and side-effects of furosemide in neonates to provide a critical, comprehensive, authoritative, and updated analysis of literature.

## 2. Bibliographic Search

The bibliographic search was performed electronically using PubMed and EMBASE databases as search engines; January 2013 was the cutoff point. The following key words were used: “diuretics neonate”, “furosemide neonate”, “pharmacokinetics furosemide neonate”, “metabolism furosemide neonate”, “continuous infusion furosemide neonate”, “extracorporeal-membrane-oxygenation furosemide neonate”, “inhaled furosemide neonate”, “furosemide patent ductus arteriosus” “ototoxicity furosemide neonate” “furosemide nephrocalcinosis neonate” “furosemide hypercalcemia neonate” “furosemide hydrocephalus neonate” and “side-effects furosemide neonate”. The bibliography of each article was read carefully, and the selected articles were examined. The references were copied by PubMed, were pasted to the manuscript and were edited according the style of Pharmaceuticals. In addition, the books NEOFAX: a Manual Used in the Neonatal Care by Young and Mangum [7] and Neonatal Formulary [10] were consulted. The findings of the bibliographic search gave rise to 120 original articles, 29 review articles and five book chapters. The publication years of this matter ranged from 1961 to 2012.

## 3. Biological Characteristics of Neonates

### 3.1. Total Body Water and Extracellular Water in Newborn Infants

Total body water was measured in 21 newborn infants with mean body weight of 3,320 g and mean total body water was found to be 78.4%. In subjects 10–15 years old, the total body water is 57.3% [17]. The extracellular water is 44.5% in neonates and 18.7% in subjects 10–15 years old [17]. In adults, Vd of furosemide is 0.13 l/kg [5] suggesting that furosemide is mainly distributed into the extracellular water. The larger Vd of furosemide in neonates than in adults may be due to the larger extracellular water in neonates into which furosemide is distributed.

### 3.2. Extracellular Volume in Neonates

The extracellular volume was measured in 14 preterm infants, with a gestational age and a birthweight of 30.7 ± 2.4 weeks and 1,473 ± 342 g, respectively, on days 1 and 7 of postnatal age. The extracellular volume decreased from 725 ± 159 mL on day 1 to 516 ± 119 mL on day 7 (*p* < 0.001) of postnatal age [18]. Thus, the extracellular volume decreases with the neonatal maturation. The drugs that are confined into the extracellular volume reach higher concentrations on day 7 than on day 1 of postnatal age.

### 3.3. Glomerular Filtration Rate in Neonates

Nephrogenesis is completed by the end of the 34th week of gestation [19]. The kidney of a full-term neonate possesses approximately 850,000 to 1,200,000 nephrons per kidney [19], but some events during pregnancy such as growth retardation, nephrotoxic drugs administered to the mother or renal/urologic fetal malformations may negatively influence the number of nephrons [20,21,22]. Glomerular filtration rate (GFR) depends on the number of nephrons, the mean arterial blood pressure, renal plasma flow, and the intra-renal resistance [23]. GFR in neonates is 2–4 mL/min and can only be maintained due to a delicate balance between vasodilatory effects at the afferent and vasoconstrictor effects at the efferent glomerular arterioles [19,21,24,25]. In adults, GFR is 120 mL/min [26]. There is an impressive postnatal increase in GFR postnatally, as it increases with a mean of 0.19 mL/min during the 7-day period between day 3 and day 10 after birth [21].

### 3.4. Tubular Function

The ultra-filtrate is modified through re-absorption and secretion processes in the different parts of the tubular system. Secretory and absorptive tubular processes are relatively well developed at birth, postnatal maturational changes occur [27]. Preterm infants have immature glomerular and tubular functions [27]. The fractional excretion of Na^+^ is an efficient index of tubular function [28]. The fractional excretion of Na^+^ directly after birth can be as high as 5% [29]. In premature infants, the fractional excretion of Na^+^ value correlated negatively with the postnatal age and the velocity of decrease was directly correlated with age [29]. In fullterm neonates, the fractional excretion of Na^+^ falls within hours [29].

### 3.5. The Loop of Henle

Functionally, the loop of Henle plays an important part in the ability to generate a concentrated urine. The descending limb of Henle’s loop, however, is thought to be permeable to Na^+^, K^+^, Cl^−^, water and urea. Permeability to Ca^+^ appears to be low [30]. Under normal circumstances, water reabsorption occurs, and the osmolality of the tubular fluid and the concentration of urea, Na^+^ and Cl^−^ increase along the descending limb [30]. Significant passive reabsorption of Na^+^ and Cl^−^ occurs along the thin ascending limb. Reabsorption of up to 25% of filtrated Na^+^ and Cl^−^ occurs in the medullary and cortical portions of the thick ascending limb [30]. Because the thick ascending limb is relatively impermeable to water, the osmolality of the tubular fluid falls progressively as solute reabsorption occurs. Approximately 20 to 25% of the filtered Ca^+^ is reabsorbed in this nephron segment [30].

### 3.6. Renal Clearance in Neonates

Clearance of drugs in neonates is slow compared with that of adults and older children, because of the relative inefficiency of renal function and lower capacity to eliminate drugs [24]. GFR matures during infancy to approach the adult rate (6 L/h/70 kg) from 6 months of postnatal life [23,31,32,33,34]. Growth restriction has an important negative effect on the normalized weight of the kidney, on the number of nephrons, on the GFR, and on tubular function in human perinatal life [35,36,37,38,39]. Infants small for gestational age have additional impact on Cl of drugs, such as the aminoglycosides or glycopeptides that are mainly cleared through the kidney. Maturation of both amikacin and vancomycin clearance is closely aligned with GFR maturation in neonates [32,33,34]. Small for gestational age preterm neonates in the first month of life have a mean reduction of 16.2% in renal drug clearance [23]. Intrauterine growth retardation has an unfavourable impact on renal tubular function [23].

### 3.7. Urine Output in Preterm Infants

Urine output was measured by Kushnir and Pinheiro [40] in 185 preterm infants with a gestational age and a body weight of 27.8 weeks and 1083 g, respectively. Urine output (mean ± SD) was 4.2 ± 1.6 mL/kg/h.

### 3.8. Renal Glomerular and Tubular Functional and Structural Integrity in Neonates

Renal functional capacity is lower in the newborns than in adults [41]. Renal cells are not fully differentiated at birth and many of the differences in renal function seen between infants and adults should be attributed to immaturity [42]. Preterm infants have immature renal function with respect to both glomerular and tubular function [29]. Plasma creatinine concentration on day 1 of postnatal life is a poor guide to an infant’s renal function because it mainly reflects the maternal creatinine origin [43]. A more precise assessment of the renal functional capacity is made by measuring the GFR. In very low birthweight infants, GFR is only 67% of that of fullterm infants [44]. The development of tubular function lags behind that of the glomerulus [45].

Urinary excretion of high molecular weight proteins, especially albumin and immunoglobulin G (IgG), is the best marker of glomerular dysfunction [46], whereas urinary excretion of low molecular weight proteins, such as α_1_-microglobulin (α_1_M), retinol binding protein (RPB) and β_2_-microglobulin (β_2_M) are recommended as potential markers for detecting tubular dysfunction [29].

The metabolic organization of the nephron has been assessed and about 12 segments have been distinguished according to their enzymatic activities. Brush-border, liposomal, and cytosolic enzymes are excreted in the urine of healthy subjects and enzymuria is expected to rise as a consequence of cell breakdown, necrosis and increased cellular turnover [47]. Therefore, the type of enzymuria reflects the site of damage to proximal tubules. Of these urinary enzymes, the brush-border membrane enzyme leucine-aminopeptidase (LAP [48]) and the lysosomal N-acetyl-β-D-glucosaminidase (NAG [49]) were recommended as markers for investigating the structural integrity of renal proximal tubules.

Table 1 shows a significant difference between days 1 and 3 in serum creatinine and urinary IgG levels among diseased preterm newborns suggesting that a glomerular dysfunction may develop in 3 days of postnatal life. Healthy preterm newborns revealed a significant difference between days 1 and 3 with respect to urinary α_1_M level and NAG activities indicating that in 3 days of postnatal life proximal tubular reabsorption and structure improve.

**Table 1:** Glomerular function, proximal tubular reabsorption function, proximal tubular structure integrity and distal reabsorption capacity in healthy fullterm infants, healthy preterm infants and diseased preterm infants on days 1 and 3 of postnatal life. Figures are the mean ± SD and analyzed using paired t test for parametric data. From Awad *et al.* [29].

Parameters	Healthy fullterm (n = 10)		Healthy preterm (n = 10)		Diseased preterm (n = 30)	
	Day 1	Day 3	Day 1	Day 3	Day 1	Day 3
**Glomerular function**						
Cr Conc. (mg/dL)	0.79 ± 0.14	0.77 ± 0.19	0.86 ± 0.21	0.84 ± 0.16	0.81 ± 0.15	0.95 ± 0.18 ^a^
Microalbuminuria (µg/mg Cr)	197 ± 245	157 ± 120	292 ± 263	154 ± 147	332 ± 263	334 ± 363
Urinary IgG (g/mg Cr)	0.02 ± 0.05	0.003 ± 0.01			0.22 ± 0.26 ^b^	0.05 ± 0.15 ^a^
**Proximal tubular reabsorption function**						
Urinary α_1_M (g/mg Cr)	99.5 ± 84.9	64.8 ± 55.3	278 ± 235 ^c^	72.3 ± 56.7 ^a^	195 ± 117	215 ± 171 ^d^
Urinary β_2_M (µg/mg Cr)	1.56 ± 2.48	4.89 ± 7.11	3.29 ± 4.69	6.12 ± 10.0	6.29 ± 4.61	8.10 ± 9.88
Urinary RBP (µg/mg Cr)	1.11 ± 1.69	1.22 ± 1.74	1.99 ± 2.50	1.20 ± 1.02	2.71 ± 2.10	3.04 ± 2.35 ^d^
**Proximal tubular structure integrity**						
Urinary LAP (U/g Cr)	0.28 ± 0.72	0.08 ± 0.06	0.47 ± 0.90	0.20 ± 0.43	0.54 ± 0.75	0.21 ± 0.26
Urinary NAG (nmol/min/mg Cr)	133 ± 192	97.7 ± 114	407 ± 395	108 ± 210 ^a^	521 ± 582	427 ± 474 ^d^
**Distal reabsorption capacity FeNa%**						
FeNa%	1.13 ± 0.98	1.48 ± 1.38	2.84 ± 3.10	1.27 ± 1.45	4.01 ± 5.90^c^	5.65 ± 6.81 ^d^

Cr = serum creatinine concentration; IgG = immunoglobulin G; α_1_M = α_1_-microglobulin; β_2_M = β_2_-microglobulin; RBP = retinol binding protein; LAP = leucine-aminopeptidase; NAG = *N*-acetyl-β-D-glucosaminidase. ^a^*p* < 0.05, statistical significant difference between days 1 and 3 within the same group of newborns. ^b^*p* < 0.05, statistically significant difference between healthy preterm and diseased preterm newborns on day 1. ^c^*p* < 0.05, statistically significant difference between healthy fullterm and healthy preterm newborns on day 1. ^d^*p* < 0.05, statistically significant difference between healthy preterm and diseased preterm newborns on day 1.

Lower levels of urinary α_1_M were demonstrated between healthy fullterms and preterm infants on day 1 of life, suggesting that proximal tubular reabsorption improves before birth. Data of day 3 comparison between both healthy and diseased preterm infants revealed a significant difference in urinary levels of α_1_M and RBP, as well as urinary activity of NAG and FeNa indicating that an alteration in the proximal tubular reabsorption, in the proximal tubular structure and in distal reabsorption capacity may develop shortly after birth.

Results of glomerular function showed significantly higher levels of serum creatinine and urinary excretion of IgG between days 1 and 3 in diseased preterm infants and no difference was observed for creatinine serum concentration in healthy preterm and fullterm infants. The damage of glomeruli leads to an increase of the filtrated load of proteins, which could lead to destruction of the structure of the proximal tubules with loss of absorptive capacity [50].

Results of urinary IgG excretion on day 3 compared with day 1 in healthy fullterm and diseased preterm infants showed a significant decrease suggesting a very transient proteinuria, and its decrement could be due to improved renal blood flow within the first days of life.

The finding that on day 1, urinary IgG levels are significantly higher in diseased preterm infants than in healthy fullterm infants could be due to alterations of the glomerular filter that occur because of the decrease of cardiac output with or without affecting absorptive tubular function in different pathological aspects of the nephron, especially in premature infants.

### 3.9. Drug Metabolism in Neonates

Drug metabolism is divided into two phases; phase I reactions include oxidation, reduction, hydrolysis and demethylation [51]. The most important group of enzymes involved in phase I are the cytochrome P-450 (CYP) isoenzymes. Phase II includes glucuronidation, sulfation, methylation and acetylation [51]. The ontogeny of drug metabolism has been reviewed by Alcorn and McNamara [20] and by Hines and McCarver [52]. CYP content in the fetal liver is about 30 to 60% the adult values [51]. Most of the information on the drug metabolism enzyme activities during development is obtained *in vitro* with mid-gestation human fetal tissues. CYP3A7 is the most abundant isoenzymes at birth with a subsequent decrease in CYP3A7 activity most prominent during the first year of life, whereas CYP3A4 and CYP2D6 are the major contributors to drug metabolism in adults [52]. With the exception of sulfotransferase (SULT1A3) which is well expressed in mid-gestational human tissues [53,54,55], glucuronosyl transferase [54,56,57], methyltransferase [58,59] and acetyl transferase [60] are little expressed in the mid-gestation human fetal tissues. Furosemide is metabolised into an acidic compound and is conjugated with glucuronic acid [61] and glucuronyl transferase is little developed in the mid-gestation human fetal liver [54,56] and kidney [54,57].

## 4. Results

This review reports 155 studies. Table 2 summarises the pharmacokinetic parameters of furosemide in neonates. In each section of this review, the literature is cited chronologically, the first articles are the most recent and the last articles are the oldest ones.

**Table 2:** Demographic data of infants and pharmacokinetic parameters of furosemide in neonates.

Population	GA (weeks)	PNA (days)	BW (g)	n	Daily dose (mg/kg)	t_1/2_ (h)	Vd (L/kg)	Cl (mL/h/kg)	Ref.
Fullterm	34.0 ± 4.7	14.5 ± 11.1	2050 ± 794	6	1 IV	9.5 ± 4.4	0.17 ± 0.03	15.4 ± 8.4	[62]
Preterm	29.0 ± 2.0	22.0 ± 26.0	1326 ± 652	8	0.91 ± 0.34 * IV	26.8 ± 12.2	0.20 ± 0.07	6.9 ± 5.1	[63]
Fullterm	39.0 ± 1.0	6.0 ± 6.0	2432 ± 786	7	1.03 ± 0.06 * IV	13.4 ± 8.6	0.52 ± 0.42	11.8 ± 9.3	
^a^ Preterm	30.0 ± 0.8	8.5 ± 1.9	1270 ± 169	14	1 IV	19.9 ± 3.0	0.24 ± 0.03	10.8 ± 7.2	[64]
Fullterm	na	1–4 months	na	12	1 IV	7.7 ± 3.0	0.83 ± 0.01	81.6 ± 15.0	
^a^ Fullterm	35.0 ± 1.8	11.5 ± 5.9	2391 ± 290a	8	1 to 1.5 IV	7.7 ± 1.0	0.81 ± 0.12	81.6 ± 15.0	[65]
Adults	___	___	___	__	___	1.3 ± 0.8	0.13 ± 0.06	99.6 ± 34.8	[5]

Figures are the mean ± SD unless otherwise stated; * p < 0.05; a Figures are the mean ± SE; na = not available; IV = intravenously.

### 4.1. Dose of Furosemide in Neonates

Initial dose of furosemide is 1 mg/kg intravenously with slow push, or intramuscularly. The dose may increase to a maximum of 2 mg/kg per dose intravenously or 6 mg/kg per dose orally [7]. In premature infants administrate furosemide every 24 h, whereas in fullterm infants administrate furosemide every 12 h [7]. Neonatal Formulary [10] suggests giving 1 mg/kg of furosemide intravenously or intramuscularly, or 2 mg/kg by mouth, repeatable after 12 to 24 h. The drug should not be given more than once every 24 h to infants with postmenstrual age of less than 31 weeks. Patients on long-term treatment with furosemide may require 1 mmol/kg per day of oral potassium chloride to prevent hypokalemia [10]. It has been suggested to giving a single 5 mg/kg furosemide intravenously for renal failure as soon as renal failure is suspected to lower the metabolic activity of the chloride pump, minimise the risk of ischemic tubular damage, and reduce the shut down in glomerular blood flow that follows from this [10]. For chronic lung disease give 1 mg/kg furosemide by nebuliser once every 6 h which may at least temporarily improve lung compliance and therefore tidal volume in some ventilator-dependent infants without affecting renal function [10].

### 4.2. Renal Response to Furosemide in Neonates

The response to furosemide can be evaluated by studying the dose response relationship between the logarithm of the urinary furosemide excretion rate and diuretic/natriuretic response [66]. Mirochnick *et al.* [67] observed a relationship between the logarithm of the urinary furosemide excretion rate and both the urinary and sodium excretion rate and urine output following initial and chronic multiple doses. There was a significant increase in the mean furosemide excretion rate associated with the midrange responses after 1 and 3 weeks of therapy compared with the initial dose. The urinary excretion of furosemide was 219 ± 130 (week 1), 959 ± 381 (week 2) and 738 ± 323 µg/kg/12 h (week 3) [67].

### 4.3. Metabolism of Furosemide in Neonates

The metabolism of furosemide in neonates was studied by Aranda *et al.* [61]. Furosemide is metabolised into an inactive acidic metabolite (2-amino-4-chloro-5-sulfamoyl anthranilic acid) and is conjugated with glucuronic acid to give inactive furosemide glucuronide. Following a 1 mg/kg intravenous dose of furosemide in newborn infants, the most rapid excretion of furosemide and its metabolites occurred during the first six hours after the dose. Mean fractions of the total urinary excretion as unchanged furosemide ranged between 52.5 and 55.6% [61]. The mean fractions of total urinary excretion as furosemide glucuronide and acidic metabolite ranged from 13.3 to 23.2% and from 20.9 to 29.3%, respectively [61]. An inverse relationship was observed between the urinary excretion of the acidic metabolite (r = −0.75; *p* < 0.05) and the excretion of unchanged furosemide and the excretion of furosemide glucuronide (r = −0.76; *p* < 0.05) and the excretion of unchanged furosemide.

### 4.4. Binding of Furosemide to Neonatal Plasma Proteins

To evoke a pharmacological effect, the drugs must leave the blood, accumulate into the tissues and bind to their receptors. In blood, drugs are bound to plasma proteins and only the unbound fraction (free fraction) may leave the blood and penetrate into the tissues. The main plasma proteins binding the drugs are albumin and α-1-acid glycoprotein [68]. Furosemide binds to albumin [69,70]. Variations in the concentration of plasma albumin yield variations in the concentrations of the unbound fraction of drugs with consequent variations in the amounts of drugs that penetrate into the tissues. In the umbilical cord plasma, the concentration of albumin ranged from 24.6 to 57.3 g/L (mean ± SD = 42.7 ± 6.5 g/L; n = 56), the coefficient of variation was 15% [71]. In adults, the concentration of albumin was 50.4 ± 6.0 g/L (n = 31; age 34 ± 6 years) and was significantly (*p* < 0.001) greater than that in the cord plasma [71]). The unbound fraction of furosemide in the umbilical cord plasma was 2.5 ± 0.1 and in adult plasma was 1.7 ± 0.7 (*p* < 0.001; [72]).

The serum concentration of albumin was measured in 13 children, aged from birth to 8 months of age, and a correlation (r = 0.763; *p* < 0.001) was found between the serum concentration of albumin and the child age [72]. The unbound fraction of furosemide was measured in the plasma of 51 children aged between 2 weeks and 13.5 years. The unbound fraction of furosemide decreased gradually from birth, approaching the adult values within the first year of life. There was a significant correlation (*p* < 0.001) between the percentage of unbound fraction of furosemide and the first six months of life [72].

Bilirubin displaces furosemide from albumin and the effect is greater in newborn infants than in adult subjects [73,74]. The unbound fraction of furosemide in the plasma of newborn infants was 2.32 ± 0.14, 2.94 ± 0.26 and 3.52 ± 0.38 in the absence, and in presence of 200 µM and 400 µM bilirubin, respectively [73]. The unbound fraction of furosemide was significantly greater in the plasma to which bilirubin was added (*p* < 0.001). The dialysis of the cord serum increased the binding of furosemide suggesting that the cord serum contains dialyzable compounds that interfere with the binding of furosemide [75]. The binding defect of furosemide in newborn plasma reflects both the effects of the endogenous inhibitors and the lower albumin concentration.

### 4.5. Pharmacokinetics of Furosemide in Neonates

Administered drugs are eliminated from the body, and the rate of drug elimination determines the concentrations of drugs in plasma and tissues. Elimination of a drug consists in two processes, metabolism and renal excretion. t_1/2_ is the time that a drug requires to halve its concentration. Vd is the apparent volume into which a drug distributes in the body at equilibrium and is an index of drug accumulation into solid tissues. Cl is the product of the constant of elimination (Ke) multiplied by Vd. Ke = 0.693/t_1/2_, thus, Cl = Vd × 0.693/t_1/2_. As t_1/2_ shortens Cl increases, postnatal maturation shortens t_1/2_ and increases Cl.

The pharmacokinetics of furosemide in neonates consists in 5 articles [6,62,63,64,65] published between 1978 and 1988. The pharmacokinetic parameters of furosemide in the newborn infants are summarised in table 2. In adults, t_1/2_, Cl and Vd of furosemide are 1.3 ± 0.8 h, 99.6 ± 34.8 mL/h/kg and 0.13 ± 0.06 L/kg, respectively [5]. In infants, t_1/2_ is 6 to 20-fold longer, Cl is 1.2 to 14-fold smaller and Vd is 1.3 to 6-fold larger than the adult values. This body of knowledge is consistent with the view that the elimination of furosemide is remarkably slower and variable in neonates than in adults and is associated with its prolonged diuretic and saliuretic effects [76,77].

The reason for prolonged t_1/2_ of furosemide in newborn infants is the slow renal excretion, related to immature renal function, which is compounded by reduced metabolic elimination. The kidney maturation drives furosemide pharmacokinetics. The capacity for non-renal elimination of furosemide must be presumed to develop at some time between birth and adulthood [62]. Aranda *et al.* [61] obtained different results, they observed that furosemide is metabolised in neonates. These authors found that furosemide is conjugated with glucuronic acid and the acidic metabolite 2-amino-4-chloro-5-sulfamoyl anthranilic acid is formed. At 6 h after administration, the urinary fractions of the parent compound, the glucuronide conjugate and the acidic metabolite were 55.4 ± 5.5%, 23.3 ± 5.5% and 21.2 ± 8.3%, respectively [61].

The postnatal development of furosemide pharmacokinetics was studied by Mirochnick *et al.* [6]. These authors administered 1 mg/kg furosemide intravenously to 10 preterm infants with a gestational age, a postnatal age and a body weight of 26.6 ± 2.9 weeks, 2.4 ± 1.0 days and 829 ± 217 g, respectively. Subsequent doses of either 1 mg/kg administered intravenously, or 2 mg/kg administered enterally, were given every 12 h to the first seven patients and every 24 h to the last three patients. Plasma t_1/2_ ranged from 1.8 to 63 h and was longest in the most immature infants. Patients < 31 weeks postmenstrual age frequently had t_1/2_ in excess of 24 h. By 33 weeks postmenstrual age, t_1/2_ had declined to less than 12 h in all infants. As postmenstrual age approached term, t_1/2_ declined to approximately 4 h. There was a remarkable interindividual variability in the kinetic parameters of furosemide in neonates. Plasma t_1/2_ of furosemide was measured in 14 preterm infants and a poor, but significant inverse correlation (r = −0.360), was found with postmenstrual age and postnatal age (r = −0.482) whereas it did not correlate with gestational age or birthweight [64]. These authors found a correlation between Cl and postnatal age (r = 0.79).

Renal Cl of furosemide ranged from 1.62 to 73.8 mL/h/kg and was lowest in the most immature infants [6]. Renal Cl increased gradually until 32 weeks postmenstrual age; after 32 weeks, renal Cl increased rapidly. The fraction of total renal Cl due to tubular secretion was small when t_1/2_ was prolonged. When t_1/2_ was >24 h, tubular secretion accounted for 13.3 ± 19.2% of total Cl. When t_1/2_ was <12 h, the secretory fraction increased to 83.7 ± 9.3% [6]. When furosemide was administered every 12 h, this drug accumulated in plasma and the highest concentration was 21.1 µg/mL. There was an inverse significant correlation (r = −0.94; *p* < 0.002) between postmenstrual age and maximum plasma concentration attained.

Bioavailability of furosemide was determined in 3 patients at a mean postmenstrual age of 39.1 weeks. Mean bioavailability was 84.3% (range 56% to 106%) [6]. Mean Vd after the initial dose was 0.23 ± 0.04 l/kg. Although Vd did not change significantly during the first 2 weeks of therapy, it did increase over the course of the entire study. Three patients, >6 weeks after the initiation therapy, received a total of 4 intravenous doses of furosemide. Mean Vd increased to 0.48 ± 0.14 l/kg, a significant (*p* = 0.0003) increase compared with Vd after the initial dose. Vert *et al.* [63] and Peterson *et al.* [64] observed that Vd is larger in fullterm than in preterm infants. This suggests that, in fullterm infants, a greater fraction of the administered dose of furosemide is distributed in periphery.

Plasma t_1/2_, Vd and Cl showed considerable variation among infants, but plasma and urinary t_1/2_ corresponded very closely for each infant (r = 0.97; *p* < 0.005) [62]. There was also a correlation between plasma Cl and renal Cl (r = 0.96; *p* < 0.005) [62]. Between 4 and 24 h after furosemide intravenous injection, renal Cl was estimated to account for a mean of 99% of total plasma Cl. Twenty-four hours after administration, mean urinary recovery of unchanged furosemide was 90% of the injected dose. The renal Cl to plasma Cl ratio did appear to decrease with increasing postnatal age (r = −0.81; *p* < 0.05; [62]). These results indicate that the renal Cl of furosemide accounts for virtually all of total plasma Cl in newborn infants. These results do not accord with those by Aranda *et al.* [61] who found that furosemide is metabolised in preterm infants. Expressed as a proportion of the intravenous injected dose, total urinary furosemide recovered was (mean ± SE) 89.7 ± 15.2% [62] and 84.4 ± 14.9% [65]. The urine elimination t_1/2_ of furosemide was measured in 13 infants, aged from 9 days to 12 months, and ranged from 0.654 to 3.29 h [78].

### 4.6. Continuous *versus* Intermittent Intravenous Infusion of Furosemide in Neonates and Infants Undergoing Cardiac surgery

Appropriate maintenance of the patient’s volume status is an important part of the perioperative management of patients undergoing cardiac surgery [79,80,81]. Patients often become volume overload after surgery, due in part to the large amounts of fluids received intra-operatively for cardiopulmonary bypass and hypotensive episodes [80]. If the hypervolemia is not corrected, it can contribute to the development of postoperative hypoxia, tissue edema, and delayed postoperative recovery [80,81]. One of the mainstays for postoperative fluid overload is the administration of furosemide [79].

The efficacy of a continuous intravenous infusion of furosemide in pediatric patients following cardiac surgery was first investigated by Singh *et al.* [82]. Infants were randomized to receive a continuous intravenous infusion or intermittent intravenous bolus doses of furosemide. The continuous intravenous infusion group received an intravenous initial furosemide dose of 0.1 mg/kg followed by an infusion rate of 0.1 mg/kg/h of furosemide to be doubled every 2 h to a maximum of 0.4 mg/kg/h if the urine output was less than 1 mL/kg/h.

The intermittent group received 1 mg/kg of furosemide intravenously every 4 h to be increased by 0.25 mg/kg every 4 h to a maximum of 1.5 mg/kg intravenously if the urine output was less than 1 mL/kg/h. The results of the trial showed no significant difference in urinary output between the 2 groups over 24 h period. A significantly (*p* = 0.045) lower daily dose of furosemide (4.90 ± 1.78 *versus* 6.23 ± 0.62 mg/kg/day) in the continuous intravenous infusion group produced the same 24-hour urine volume as that of the intermittent group. There was more variability in urine output in the intermittent group as well as more urinary losses of sodium (0.29 ± 0.15 *versus* 0.020 ± 0.06 mmol/kg/day; *p* = 0.007), and chloride (0.40 ± 0.20 *versus* 0.30 ± 0.12 mmol/kg/day; *p* = 0.045).

Three cases of supraventricular tachycardia, associated with the use of furosemide infusion following cardiac surgery, were reported by Wilson *et al.* [83]. The supraventricular tachycardia occurred 3 to 7 h after starting an intravenous infusion of furosemide at 1.0 mg/kg/h. All three patients had a diuresis of 8 to 10 mL/kg/h compared with a mean average of 2.5 mL/kg/h in 22 other patients who had received a similar infusion. A rapid fluid shift was the most likely mechanism of the tachycardia. Sotalol was effective in controlling the tachycardia in the two patients in whom it was tried. These authors recommend a starting dose of 0.3 mg/kg/h in infusing intravenously furosemide, with hourly increments of 0.1 mg/kg/h until the desired diuresis have been obtained.

Luciani *et al.* [84] compared the safety and efficacy of continuous *versus* intermittent intravenous furosemide. Continuous infusion of furosemide was administered to 11 infants with an age and a body weight of 3.7 ± 3.4 months and 4.6 ± 2.1 kg, respectively. The patients were given an initial dose of 0.1 mg/kg intravenous furosemide and then continuous infusion was started at 0.1 mg/kg/h. This infusion rate was doubled every 2 h (to a maximum of 0.4 mg/kg/h) if the urinary output was less than 1 mL/kg/h in the 4 previous hours of observation. Intermittent furosemide was administered to 15 infants with an age and a body weight of 1.8 ± 2.5 months and 4.3 ± 1.7 kg, respectively. The patients were given 1 mg/kg of furosemide intravenously every 4 h. The dose was increased by 0.25 mg/kg, to a maximum of 2 mg/kg intravenously, if the urine output was less than 1 mL/kg/h in the previous 4 h of observation. The efficacy of furosemide in terms of urinary output was satisfactory in both patient groups. When the urinary volume was corrected for the dose of drug administered, a significantly larger response could be demonstrated with continuous intravenous infusion. The dose of furosemide administered was 2.5 ± 0.3 (continuous infusion) and 6.8 ± 12 mg/kg/day (intermittent infusion; *p* = 0.001). Urinary output per dose of drug was significantly larger after continuous intravenous infusion (1.0 ± 0.4 mL/kg/h) than after intermittent intravenous furosemide (0.5 ± 0.2 mL/kg/h; *p* = 0.002) with lesser fluctuations (variance, 1.9 ± 1.6 *versus* 3.8 ± 2.1; p = 0.02) and fluid replacement needs (20.6 ± 3.8 *versus* 51.8 ± 14.4; p = 0.0017). Luciani *et al.* [84] conclude that commonly used doses of both intermittent and continuous furosemide infusion can be safely administered to critically ill neonates and infants as early as 6 h after operation.

Intermittent intravenous therapy with furosemide may cause unpredictable fluctuations in the serum concentrations of the drug, thereby exposing the patients to the risk of ototoxicity and nephrotoxicity [85,86].

There are a number of critically ill infants in whom diuretic response to conventional furosemide administration may be suboptimal or even absent. These patients are generally considered “diuretic resistant” [66].

In a study by Copeland *et al.* [87] on 18 adult patients who were hemodynamically stable after cardiac surgery, no significant difference was demonstrated in terms of urinary output between continuous intravenous infusion and intermittent intravenous administration of furosemide. Continuous intravenous infusion, however, offered a more constant urinary flow.

Klinge *et al.* [88] compared the urine output in 46 children undergoing cardiac surgery following intermittent intravenous or continuous intravenous administration. Twenty-three children aged 2.4 ± 2.1 years with a body weight of 10.8 ± 5.7 kg received intermittent intravenous furosemide and other 23 infants aged 3.4 ± 3.1 years with a body weight 14.5 ± 9.3 kg received continuous intravenous infusion of furosemide. The initial dose of intermittent intravenous furosemide was 0.5 mg/kg per day, and the initial dose of continuous intravenous infusion of furosemide was 2 mg/kg per day. The patients in the intermittent group needed a significantly lower daily dose of furosemide.

When the dose of furosemide was adjusted to the patient’s need, instead of administering in a fixed dose as it had been in the studies by Copeland *et al.* [87], van Meyel *et al.* [89] and Lahav *et al.* [90], intermittent intravenous administration of furosemide resulted in a significantly lower daily dose of furosemide over the study period of 3 days and the dose was significantly more effective.

Vanpeè *et al.* [91] observed that the development of glomerular and tubular renal function is delayed in preterm infants. van der Vorst *et al.* [92] evaluated the pharmacokinetics and effects of continuous intravenous furosemide in hemodynamically unstable pediatric patients after cardiac surgery. Nineteen infants with a median age of 13 weeks (range 0 to 33 weeks) and a body weight of 4.2 kg (range 3.0 to 6.6 kg) requiring a 3-day treatment of continuous intravenous infusion of furosemide were enrolled in the study. Ten patients received one or more furosemide boluses (mean ± SD, 1.4 ± 1.1) and the bolus doses had a mean of 1.3 ± 0.4 mg/kg. The furosemide intravenous infusions were started at a median time of 30 h after cardiac surgery with a rate of 0.1 mg/kg/h. The median urinary furosemide excretion rate at the end of day 1, 2 and 3 was 0.45, 0.70 mg/h, and 0.69 mg/h, respectively. The median urinary sodium excretion was 2.1, 9.4, and 11.5 mmol/kg per 24 h over days 1, 2 and 3, respectively. The median urinary output was 2.4, 5.8, and 5.4 mL/kg/h over the respective treatment days.

Continuous intravenous infusion of furosemide is superior to intermittent infusion of furosemide, because continuous intravenous administration results in a more controlled diuresis [82,84,88,93]. van der Vorst *et al.* [93] studied 15 infants with a median of 12 weeks (range 0.2 to 35 weeks) and the weight was 4.0 kg (range 3.0 to 6.2 kg) undergoing cardiopulmonary bypass surgery who were hemodynamically unstable and in whom transient renal insufficiency occurred. At the start of the study, nine patients were diagnosed with acute renal failure. The total dose of furosemide boluses administered to the infants before the start of the continuous infusion was 2.94 ± 1.08 mg/kg. Continuous intravenous infusion of furosemide was started at a median time 25 h (range 14 to 34 h) postoperatively. The mean continuous intravenous furosemide dose was 0.22 ± 0.06, 0.25 ± 0.10 and 0.22 ± 0.11 mg/kg/h on the first, second and third day, respectively. There was a strong linear relationship between sodium excretion and urine production, with correlation coefficients ranging from 0.66 to 0.99 (p ranged from 0.02 to 0.0003). With increasing urinary furosemide excretion, higher values of sodium excretion and urine output were observed, with no indication of tolerance.

Van der Vorst *et al.* [94] stated that tolerance of furosemide effect does not develop with prolonged diuretic exposure. It has been suggested that the tolerance to furosemide can be induced through different but complementary homeostatic mechanisms in the kidney [95,96]. The mechanism underlies the development of tolerance is dehydration. This provides a possible explanation as to why tolerance was not observed in the patients, because they were volume overloaded and certainly not dehydrated at any time during the continuous furosemide infusion. There are no indications that tolerance develops towards the diuretic effect of furosemide in hemodynamically unstable infants with volume overload after cardiac surgery with cardiopulmonary bypass, who are treated with a relatively aggressive diuretic regimen with furosemide. It is unlikely that the high furosemide dose was associated with renal toxicity [94]. Relatively high furosemide doses did not result in metabolic alkalosis. These authors conclude that the employed furosemide regimen can be used safely in haemodynamically unstable infants after cardiac surgery.

### 4.7. Furosemide in Neonates and Infants Treated with Extracorporeal Membrane Oxygenation

Extracorporeal membrane oxygenation (ECMO) is a modified heart-lung machine combined with a membrane oxygenator to provide cardiopulmonary support for patients with reversible pulmonary failure in whom conventional therapies failed [97]. A 20-year experience on neonatal ECMO was reported by Schaible *et al.* [8]. Randomized studies showed improved survival for neonates treated with ECMO in comparison with conventional treatment [98,99]. During the period 1987 to 2006, 321 newborns with respiratory failure were treated with ECMO [8]. Mean gestational age was 38.7 weeks, and mean birthweight was 3163 g. diaphragmatic hernia was the most common diagnosis (53%), followed by meconium aspiration syndrome (21%), sepsis and/or pneumonia (13%) and others (13%).

Van der Vorst *et al.* [100] explored a continuous intravenous furosemide regimen that adapts to urine output in neonates treated with ECMO. Seven neonates admitted to a pediatric surgical intensive care unit for ECMO therapy, younger than 1 year of age, were enrolled in the study. The continuous intravenous furosemide infusion was started at a rate of 0.2 mg/kg/h and was preceded by a loading bolus of 1 mg/kg in patients with normal renal function and 2 mg/kg in patients with acute renal failure. The aim was to reach and maintain a urine output of 6 mL/kg/h.

The volume of ECMO circuit was approximately 400 mL, and the solution consisted of albumin and packed red blood cells. The mean ± SD of furosemide dose was 0.17 ± 0.06 mg/kg/h, 0.08 ± 0.04 mg/kg/h, and 0.12 ± 0.07 mg/kg/h, respectively, over the first, second and third day of the study. Furosemide Vd was 0.5 l/kg (range 0.2 to 2.7 l/kg). Furosemide concentration, 10 min after the loading bolus, was 1.95 µg/mL (range 0.4 to 4.7 µg/mL), and the concentration in all of the samples (n = 15) taken during the entire observation period was 3.1 µg/mL (range 0.4 to 12.9 µg/mL).

Urine production from the start of ECMO until the start of furosemide therapy was 2.2 mL/kg/h, and increased to 7.9 mL/kg/h and 6.1 mL/kg/h after 8 and 16 h, respectively, of continuous intravenous furosemide infusion. The median urine production over the consecutive 3 days was 6.8 mL/kg/h, 6.0 mL/kg/h, and 5.4 mL/kg/h. The target urine production was reached after a median time of 7 h (3 to 37 h). Followed by a continuous infusion at 0.2 mg/kg/h, which was adjusted according to the target urine production of 6 mL/kg/h, the mean ± SD furosemide dose was 0.17 ± 0.06 mg/kg/h, 0.08 ± 0.04 mg/kg/h and 0.12 ± 0.07 mg/kg/h, respectively, on the first day, second day and third day of the study. The median urine production over the consecutive study days was 6.8 mL/kg/h, 6.0 mL/kg/h and 5.4 mL/kg/h [100]. The regimen was hemodynamically well tolerated and the median furosemide serum concentrations were 3.1 µg/mL, well below the toxic level. Furosemide infusion appears an effective means to reduce volume overload in neonates treated with ECMO.

Van der Vorst *et al.* [9] evaluated the furosemide regimen in neonates treated with ECMO. Thirty-one infants with a mean gestational age of 40 weeks and a mean postnatal age of 1 day (range 0 to 16 days) and median body weight of 3.5 kg were enrolled in the study. Prior to the start of continuous intravenous infusion of furosemide, seven patients received an intravenous furosemide bolus (0.4 to 2.4 mg/kg). The continuous intravenous furosemide dose, in the patients who received a bolus prior to the infusion, was 0.08 mg/kg/h (range 0.04 to 0.13 mg/kg/h); in the patients who did not receive a bolus, the dose was 0.08 mg/kg/h (range 0.02 to 0.17 mg/kg/h) mg/kg/h. Mean urine production remained 6.2 mL/kg per 24 h of continuous infusion of furosemide in all patients irrespective of a bolus prior to the continuous furosemide infusion.

The dosing schedule of continuous intravenous infusion of furosemide in neonates treated with ECMO is largely empirical because of the variable renal function and the altered furosemide pharmacokinetics. van der Vorst et al [9] observed that the dose of the continuous intravenous infusion of furosemide varied from 0.02 to 0.17 mg/kg/h, and that 39% of the patients received additional diuretic. Although the urine output was satisfactory in patients studied, the use of additional diuretic suggests that the applied infusion rates were not optimal.

The limited studies conducted in infants receiving ECMO have identified significant differences in pharmacokinetic parameters. These differences arise from a complex interplay of changes in physiology resulting from the addition of ECMO circuit, the patient’s underlying dynamic disease process, and the alteration caused by disruptions in blood flow and drug binding to the circuit [101]. Furosemide was substantially adsorbed onto ECMO circuits components. Analysis of four ECMO circuits into which doses of 5 and 10 mg furosemide were injected revealed a 63 to 87% reduction in serum furosemide concentrations measured over 4-hour observation period. This loss of drug was most pronounced within the first 30 min of distribution through the circuit [101].

### 4.8. Inhaled Furosemide in Preterm Infants

Kugelman *et al.* [102] hypothesized that inhaled furosemide will result in improved pulmonary mechanics in ventilated infants with bronchopulmonary dysplasia and will prevent the systemic complications of parenteral furosemide. A randomized, double-blind, crossover study was performed on 9 infants with bronchopulmonary dysplasia, each serving as his own control. The infant gestational age was 29 ± 1 weeks, the birthweight was 1100 ± 100 g, the age at study was 47 ± 6 days, and weight at study was 1800 ± 200 g. Each patient was randomized to receive an aerosol dose of furosemide (1 mg/kg in 2 mL of saline) or placebo (2 mL of saline). Pulmonary mechanics were measured before and 1 and 2 h after the inhalation using the “Pulmonary Evaluation and Diagnostic System”. There was no significant change in dynamic compliance, dynamic resistance, and tidal volume 1 or 2 h after treatment with either furosemide or placebo. Kugelman *et al.* [102] concluded that, under the conditions of study, a single dose of 1 mg/kg inhaled furosemide does not improve the pulmonary mechanics in ventilator-dependent infants with severe bronchopulmonary dysplasia.

Bar *et al.* [103] evaluated the short- and long-term effects and the treatment feasibility of inhaled furosemide as compared with placebo via hood in hospitalised infants with viral-bronchiolitis. A randomised, double-blind, placebo-controlled study was performed with 16 infants in each group; postnatal age was 72 ± 43 days. Enrolled infants were randomized to receive either inhaled-furosemide (2 mg/kg), or placebo nebulised by hood three times daily throughout the hospitalisation. Clinical assessment (Respiratory Distress Assessment Instrument) was performed, 30 and 60 min after the first daily inhalation. The short-term effects were evaluated by “respiratory distress assessment instrument”, and the long-term effects by time to be weaned off oxygen, time to full enterally feeding, length of stay, and “ready to discharge” time. Oxygen requirement decreased significantly at 30 min post-inhalation only in the inhaled furosemide group. Respiratory assessment change score and long-term effects of both groups were comparable. These results showed that inhaled furosemide has no significant clinical effects in hospitalized infants with viral-bronchiolitis.

Prabhu *et al.* [104] compared the effects of a single dose of furosemide (1 mg/kg) administered either intravenously or by nebulisation on pulmonary mechanics in 19 premature infants, at 24 to 30 weeks of gestational age, evolving chronic lung disease. The infant mean postnatal age was 23 days (range 14 to 52 days). Infants had been dependent on mechanical ventilation since birth. Furosemide was administered, in random order, intravenously and by nebulisation, on two separate occasions 24 h apart. Pulmonary function studies were performed before and at 30, 60 and 120 min after administration of furosemide. Nebulised furosemide increased the tidal volume 31 ± 11.5% and compliance 34 ± 12% after two hours, whereas no change in either was noted for up to two hours after intravenous furosemide administration. Neither intravenous nor nebulised furosemide had any effect on airway resistance. Prabhu *et al.* [104] conclude that a single dose of nebulised furosemide improves pulmonary function in premature infants with evolving chronic lung disease.

Ohki *et al.* [105] investigated whether aerosolized furosemide would improve pulmonary function in infants with chronic lung disease. Eight preterm ventilator-dependent infants were enrolled in a cross-over, double-blind, placebo-controlled study. Either aerosolized furosemide (2 mg/kg) or placebo (0.9% saline) was administered, and serial pulmonary function tests were performed before and at 1 and 2 h after each inhalation. After furosemide inhalation, static respiratory compliance increased significantly by 24.3% and 23.2% as percentage change from the baseline value at 1 and 2 h (*p* = 0.014 and 0.022, respectively). Also tidal volume increased significantly by 33.8% and 28.7% at 1 and 2 h, respectively (*p* = 0.004 and 0.009). In contrast, no changes were observed in the infants after placebo inhalation. Total respiratory resistance was unchanged after both furosemide and placebo inhalation. These data suggest that aerosolized furosemide improved pulmonary function in infants with chronic lung disease without excessive diuresis.

Rastogi *et al.* [106] studied the effects of different doses of nebulised furosemide on 8 preterm infants with bronchopulmonary dysplasia who were supported by mechanical ventilation. Doses of 1 mg/kg furosemide significantly improved lung compliance (51% at 2 h after nebulisation), pulmonary resistance (28% at 1 h), and tidal volume (43% at 1 h), starting as early as 30 min after the dose. The effect lasted for at least 4 h in many of the infants and was not associated with diuretic or renal side-effects.

Belik *et al.* [107] prospectively evaluated the effect of early furosemide-induced diuresis in 39 neonates less than 24 h of age with clinical respiratory syndrome who received either 4 doses of furosemide (1 mg/kg) or no diuretic. The furosemide group overall showed a significant decrease (*p* < 0.01) in alveolar-arterial oxygen gradient, and peak inspiratory pressure (32 h *versus* 52 h) accentuated in the subgroup with 1,000 to 1,500 g birthweight, while no increase in urine output was observed for the infants weighing <1,000 g. A significant reduction in supplemental oxygen and need for ventilatory support at 96 h of age was observed in the furosemide-treated, less than 1,500 g infants. The results by Blik *et al.* [107] suggest that early furosemide-induced diuresis, particularly in infants weighing 1,000 to 1,500 g at birth, promotes improvement in pulmonary functions in respiratory distress syndrome and leads to faster reduction in oxygen and ventilator support.

The literature on the effects of inhaled furosemide on pulmonary mechanics is discordant. Some authors found that inhaled furosemide improves lung compliance whereas other authors found that inhaled furosemide has no significant improvement on lung mechanics. It is difficult to draw a conclusion about the efficacy of inhaled furosemide on lung function. Stewart *et al.* [11] reviewed the risk and benefits of inhaled furosemide administration in preterm infants with respiratory distress syndrome. These authors conclude that there are no data to support routine administration of furosemide in preterm infants with respiratory distress syndrome. Brion and Soll [13] assessed the risks and benefits of diuretic administration in preterm infants with respiratory distress syndrome and reached the same conclusions achieved by Stewart *et al.* [11].

### 4.9. Furosemide in Preterm Infants with a Patent Ductus Arteriosus

Furosemide stimulates the renal synthesis of prostaglandin E2 [12], a potent dilator of the ductus arteriosus. Thus, furosemide has an opposite effect to ibuprofen and indomethacin which are the drugs used to close the patent ductus arteriosus. Recently, Stewart *et al.* [11] and Brion and Soll [13] reached the conclusion that elective administration of furosemide to any infants with respiratory distress syndrome should be carefully weighed against the risk of precipitation of a symptomatic patent ductus arteriosus.

Lee *et al.* [108] identified the effects of furosemide, after indomethacin administration on the rate of patent ductus arteriosus closure and renal function, in preterm infants with a gestational age <34 weeks and a birthweight <2,000 g receiving indomethacin therapy (one course: 0.2–0.1–0.1 mg/kg every 12 h) mostly started <48 h after birth. Thirty-five infants received furosemide (1 mg/kg) and 33 infants were the controls. Each infant received indomethacin. The outcomes were the closure of patent ductus arteriosus and the presence of acute renal failure. These authors found that there were no differences in the patent ductus arteriosus closure rate between furosemide (85%) and the controls (93%; *p* = 0.437). The incidence of acute renal failure (serum creatinine >1.6 mg/dL) was greater in the furosemide group (59%) than in the control group (10%; *p* < 0.001). Lee et al [108] conclude that the use of furosemide in combination with indomethacin increased the incidence of acute renal failure but did not affect the patent ductus closure rate in preterm infants.

Brion and Campbell [109] assessed studies for possible bias and for quality of assessment of ductal patency. These authors studied whether furosemide affects the incidence of failure of patent ductal closure after indomethacin treatment. Furosemide administration did not significantly increase the risk of failure of ductal closure; however, sample size was insufficient to rule out even a 31% increase. There is not enough evidence to support the administration of furosemide to premature infants treated with indomethacin for symptomatic patent ductus arteriosus.

Green *et al.* [110] administered furosemide (1 mg/kg) to 33 premature infants with the respiratory distress syndrome, to determine whether it increased the incidence of patent ductus arteriosus. Furosemide stimulates the synthesis of prostaglandin E2 which inhibits the closure of the ductus arteriosus. Chlorothiazide, a diuretic that does not stimulate prostaglandin E2 synthesis, was used as the control drug in other 33 preterm infants. The incidence of patent ductus arteriosus was significantly higher (*p* < 0.02) in the furosemide group (18 out of 33 infants; 54%) than in the chlorothiazide group (8 out of 33; 24%). Six infants, all from the furosemide group, who did not show evidence of a patent ductus during the study were later found to have one. When the analysis was repeated after the fifth day of life, prostaglandin E2 excretion tripled after furosemide administration, whereas no increase occurred with chlorothiazide. Green *et al.* [110] conclude that furosemide increases the incidence of patent ductus arteriosus in premature infants with the respiratory distress syndrome, probably through a prostaglandin-mediated process.

Sulyok *et al.* [12] studied the effects of furosemide (1 mg/kg intramuscularly) on prostaglandin E2 and prostaglandin F2α synthesis in 19 newborn infants with a mean gestational age 38.7 weeks (range 36 to 41 weeks) and with a mean birthweight of 3,009 g (range 2,700 to 4,150 g) at the postnatal age of 4 to 7 days. After the administration of furosemide, urinary prostaglandin E2 increased from 17.53 ± 3.37 to 23.73 ± 3.16 ng/12 h (*p* < 0.025) and prostaglandin F2α increased from 16.48 ± 4.12 to 26.27 ± 4.12 ng/12 h (*p* < 0.05). These authors concluded that 1 mg/kg furosemide increases the urinary excretion of prostaglandin E2 and prostaglandin F2α in newborn infants.

Friedman *et al.* [111] measured the urinary excretion of prostaglandin E2 in 7 sick low-birthweight infants. After 1 mg/kg furosemide, prostaglandin E2 excretion rate increased from 0.4 ± 0.04 to 1.3 ± 0.2 ng/mg creatinine. Following the administration of indomethacin to 2 patients with patent ductus arteriosus, the urinary excretion of prostaglandin E2 decreased. These results demonstrate that furosemide enhances urinary excretion of prostaglandin E2 by mechanisms which may reflect an increase in prostaglandin synthesis. Indomethacin, which is a prostaglandin synthetase inhibitor, decreases the urinary excretion of prostaglandin E2.

Yeh *et al.* [112] investigated if furosemide would prevent the renal side-effects of indomethacin therapy in premature infants with patent ductus arteriosus. Nine premature infants received indomethacin alone, and ten premature infants received indomethacin followed immediately by furosemide. Infants who received indomethacin and furosemide had significantly higher urine output (*p* < 0.05) and higher GFR (*p* < 0.05) than those of infants who received indomethacin alone. Seven infants in each group responded to indomethacin therapy with the disappearance of patent ductus arteriosus murmur which prevented the renal side-effects of indomethacin therapy and yet did not affect the efficacy of indomethacin in the closure of the patent ductus arteriosus.

### 4.10. Ototoxicity Following Furosemide Administration to Adults and Neonates

Little is known about the ototoxicity due to furosemide administration to neonates and most of the information deals with adult patients. Schwartz *et al.* [113] reported transitory hearing loss in five adult patients who received high doses of furosemide intravenously. Heidland and Wigand [14] found that among patients with severe renal failure, infusion of furosemide at a constant rate of 25 mg/min caused noticeable hearing loss in two thirds of their patients. When the infusion rate was reduced to 15 mg/min, only minor hearing losses were reported. These authors concluded that furosemide should be given at a rate less than 4 mg/min to adult patients to avoid hearing loss. Morelli *et al.* [114] studied the effects of furosemide in ten adult patients with chronic renal failure. The patients were given 2,000 mg of furosemide in 200 mL of isotonic saline over 30 min period. This resulted in tinnitus and deafness which lasted about 90 min in all patients who recovered normal hearing later. Fries *et al.* [115] found no hearing loss among adult patients with renal failure who received 500 or 1,000 mg of furosemide by infusion over six hours. Rastogi *et al.* [116] reported no episodes of hearing loss in patients receiving oral doses of furosemide up to 2 g daily. Some authors [117,118,119] have reported permanent hearing loss following smaller oral doses of furosemide. Wigand and Heidland [120] reported audiometric studies in patients receiving furosemide. They found that rapid infusion of high doses of furosemide (1 g in 40 min) produced acute hearing loss in half of the patients studied. These reversible hearing losses were greatest in the middle frequency range. Although most cases of furosemide ototoxicity have been reversible, some authors [117,118,119,121,122] have reported permanent deafness after furosemide administration. Kshirsagar *et al.* [123] reported a case of hearing loss following 25 mg/kg of furosemide infused slowly intravenously over 4 h in a patient with nephritic syndrome.

Among 547 preterm infants of ≤34 weeks of gestation, eight infants (1.46%) developed severe progressive and bilateral sensorineural hearing loss [124]. Perinatal risk factors of infants with hearing loss were comparable with those of a control group matched for gestational age, birthweight and perinatal complications. There was an association of hearing loss with higher incidence of perinatal complications. Ototoxicity appeared closely related to a prolonged administration and higher total dose of ototoxic drugs, particularly aminoglycosides and furosemide. Borradori *et al.* [124] strongly recommend prospectively and regularly performing audiologic assessment in sick preterm infants as hearing loss is of delayed onset and in most cases bilateral and severe.

The Neonatal Formulary [10] states that concurrent furosemide use significantly increases the risk of aminoglycosides ototoxicity. Aminoglycosides should not be administered in association with furosemide. Methods of avoiding ototoxicity include slow continuous infusion rather than bolus injection, use of divided oral dose regimens, and the measurement of blood levels to avoid exceeding 50 µg/mL of furosemide [125]. If a diuretic response cannot be obtained using the above measures, the substitution of another diuretic such as bumetanide is suggested to maintain the therapeutic response and minimize the ototoxicity [125].

Rybak *et al.* [126] investigated the effects of furosemide on cochlear function and the stria vascularis ultrastructure in rats of various postnatal ages. After an intravenous injection of furosemide (35 mg/kg) to anesthetized rats, the endocochlear potential and compound action potential of the eighth nerve were recorded. Rat pups 9 to 28 days of age had much greater reduction of endocochlear potential and elevation of the compound action potential threshold than animals older than 30 days. These findings support the concept of a critical period of susceptibility to ototoxic drugs during development and could have important clinical implications in premature infants.

### 4.11. Furosemide and the Risk for Nephrocalcinosis in Preterm Infants

Infants with low birthweight treated with chronic furosemide therapy are at risk for the development of intra-renal calcifications [16]. The pathogenesis of nephrocalcinosis in very low birthweight infants appears to be multifactorial. The vulnerability of extreme immaturity and the underdevelopment of renal function may be the most important variables. Hypercalciuria is common in very low birthweight infants, yet not all develop nephrocalcinosis. Decreased GFR, low citrate excretion, and frequently an alkaline urine are in part due to the immaturity of renal function of these infants. The bronchopulmonary dysplasia, frequently requiring furosemide that may cause phosphaturia and magnesium depletion, and that may increase calcium excretion, are more common in the smallest and sickest of premature infants [16].

Giapros *et al.* [127] estimated renal glomerular and tubular functions and kidney length during the 2 years of life of preterm infants with nephrocalcinosis associate with prematurity. The study cohort comprised 63 infants with nephrocalcinosis and 44 control subjects who were matched for gender, gestational age and birthweight. These authors concluded that nephrocalcinosis in the preterm infants was associated with impaired renal tubular function and a shorter kidney length in the first year of life.

Nasseri *et al.* [128] determined the incidence and risk factors of nephrocalcinosis in 49 infants with a birthweight of about 1,500 g treated with furosemide. Nephrocalcinosis was observed in 13 (26.5%) infants and was bilateral in 7 (14.3%) infants. The mean age of diagnosis of nephrocalcinosis was 52.6 days (range 30–123 days). The mean duration of ventilation was significantly less in infants with than without nephrocalcinosis (*p* = 0.02), and the mean levels of urine calcium and phosphate, at 4 weeks of age, were respectively (*p* = 0.013; *p* = 0.048). Using logistic regression analysis, family history of renal stone (*p* = 0.002) and urine calcium/creatinine ratio were significant (*p* = 0.011) predictors of nephrocalcinosis. Nasseri *et al.* [128] conclude that family history of renal stone and urine calcium/creatinine ratio are the major risk factors of nephrocalcinosis in very low birthweight neonates.

Gimpel *et al.* [129] determined which of the many risks factors for nephrocalcinosis in preterm infants are most relevant. Fifty-five infants with a gestational age <32 weeks and a body median weight 1,010 g (range 500–2,070 g) were studied. In multivariate analysis, the strongest independent risk factor was furosemide therapy above 10 mg/kg cumulative dose, with 48-fold increase of nephrocalcinosis. The risk of nephrocalcinosis was 1.65-fold higher per 100 g lower weight (1.07 to 2.56; *p* = 0.02) and 4.5-fold higher per mmol/l of urinary calcium concentration (1.14 to 17.7; *p* = 0.03). These authors suggest that in preterm infants, furosemide should be prescribed with caution and close monitoring of calcium excretion is advisable.

Ketkeaw *et al.* [130] analysed 36 infants with a gestational age and a body weight less than 32 weeks and 1,250 g, respectively. Fourteen infants had abnormal ultrasound scans compatible with nephrocalcinosis giving an overall incidence of 38.9%. Factors associated with nephrocalcinosis included severity of respiratory illness, patent ductus arteriosus, oxygen dependency and furosemide therapy. Ketkeaw *et al.* [130] conclude that very low birthweight and preterm infants have a risk of developing nephrocalcinosis especially those with severe respiratory illness and prolonged use of furosemide.

Pope *et al.* [131] investigated the history of nephrocalcinosis in premature infants treated with furosemide. Thirteen preterm infants were studied. During hospitalisation and while receiving furosemide, nephrocalcinosis developed in each patient. Patients were divided into two groups based on resolution (six infants) and non resolution (seven infants) according to spontaneous resolution of nephrocalcinosis at any point during follow-up. Mean follow-up after discontinuation of furosemide in the resolution *versus* non-resolution groups was 10.3 and 7.7 months, respectively. Early data indicate that nephrocalcinosis resolves in approximately 50% of premature infants 5 to 6 months after discontinuation of furosemide. 

Nephrocalcinosis and nephrolithiasis developed in five children after furosemide therapy for congestive heart failure. Elimination of furosemide in three children resulted in resolution of the calcifications. The phenomenon of renal calcification associated with furosemide treatments is more frequent than previously recognized [132].

Downing *et al.* [133] determined the long-term renal sequelae following the use of furosemide in preterm infants. The renal function of twenty-seven preterm infants with a birthweight <1500 g was evaluated at 1 to 2 years of age. Patients were classified into three groups on the basis of status at the time of discharge from the hospital. Group 1 (n = 7) had no furosemide treatment or renal calcifications, group 2 (n = 10) had furosemide therapy but no calcification, and group 3 (n = 10) had furosemide therapy with renal calcification. No difference in renal function was observed between groups 1 and 2. Children in group 3 had significantly higher urinary calcium/creatinine ratios and fractional excretion of sodium and lower tubular reabsorption of phosphate than children in the other two groups. Downing *et al.* [133] conclude that furosemide-related renal calcifications in very low birthweight infants may lead to glomerular and tubular dysfunction; further long-term follow-up of this population is recommended.

Downing *et al.* [134] performed a prospective longitudinal renal ultrasound investigation on 117 premature infants. Of these infants, 20 had intra-renal calcifications. Eight patients at age 16.3 ± 2.6 months had sonographic resolution of renal calcifications, 6.6 ± 1.1 months after furosemide therapy had been discontinued. Twelve children, continued to receive furosemide for their chronic lung disease demonstrating significant association between chronic use of furosemide and persistence to the renal calcification (*p* < 0.001). These authors conclude that discontinuation of furosemide therapy is associated with resolution of the renal calcifications.

Jequier and Kaplan [135] reported a retrospective study of 68 infants with increased medullary echogenicity on renal ultrasound examination showed nephrocalcinosis to be present in 42 patients. The cause was believed to be iatrogenic in 30 and non-iatrogenic in 12 infants. Furosemide therapy was responsible for 11 of the cases of iatrogenic nephrocalcinosis.

Kenney *et al.* [136] treated three very low birthweight infants with furosemide for bronchopulmonary dysplasia. All infants developed medullary nephrocalcinosis identifiable by real time ultrasound. The sonographic findings of diffuse medullary hyper-echogenicity appeared to be specific for nephrocalcinosis.

Hufnagle *et al.* [137] observed that ten premature infants developed renal calcification while receiving long-term furosemide therapy. These infants received 2 mg/kg/day of furosemide for at least 12 days before calcifications were noted on abdominal roentgenograms. Calcifications included small flecks, isolated stones, staghorn calculi and nephrocalcinosis. Analysis of stone showed calcium oxalate and calcium phosphate. Infants who were not receiving furosemide had no calcifications. The infants with renal calcifications had rates of calcium excretion 10 to 20 times the normal. These authors conclude that furosemide, in doses of at least 2 mg/kg/day for at least 12 days can be associated with renal calcifications. The probable mechanism of the stone formation is hypercalciuria, primarily caused by furosemide.

### 4.12. Furosemide may Yield Hypercalcemia in Neonates

Chang *et al.* [138] searched the factors significantly associated with renal calcifications in 102 infants with very low birthweight. Only 6 infants (6%) had renal calcification at term or before discharge compared with 96 who did not. Factors associated with renal calcification included furosemide therapy (33% *versus* 3%; *p* = 0.027), and dexamethasone therapy (50% *versus* 2%; *p* = 0.001). Three of six patients had spontaneous remission of renal calcification. Chang *et al.* [138] concluded that the incidence of renal calcification in very low birthweight infants was relatively low, and the calcification was transient in one-half of the infants. Extremely premature, sick infants requiring long-term ventilation, and those receiving furosemide or dexamethasone were more likely to have calcification.

Srivastava *et al.* [139] reported two infants with parathyroid hormone-related protein hypercalcemia secondary to congenital mesoblastic. Pre-operative hypercalcemia was corrected with saline hydration, furosemide, calcitonin and/or pamidronate.

Nair *et al.* [140] reported a 30-day-old infant with subcutaneous fat necrosis and symptomatic hypercalcemia, who developed metastatic calcification in the subcutaneous tissue, kidneys, pericardium and brain. The infant had anemia, hypertriglyceridemia and hypercholesterolemia. The infant was managed with intravenous saline, furosemide, oral steroids and bisphosphonates and improved with treatment.

Pradhan and Leonard [141] reported a 5-day-old infant with severe associated hypercalcemia secondary to a solid tumor in the pelvis. Aggressive pharmacological treatment with furosemide, pamidronate, and calcitonin failed to reduce the serum calcium adequately. Implementation of calcium-free hemodialysis resulted in a rapid reduction of serum calcium from 22.6 to 11.6 mg/dL.

### 4.13. Furosemide, Acetazolamide and Hydrocephalus

Hydrocephalus is an excess accumulation of cerebrospinal fluid in or around the brain. Although the standard treatment of hydrocephalus is cerebrospinal fluid shunting, there are certain circumstances in which medical treatment, alone or in combination with shunting, has been suggested as an alternative. The incidence of development delay, cerebral palsy, epilepsy and visual impairment in surviving children is variable. The most common treatment of posthemorragic hydrocephalus involves permanent ventricular shunting. Conservative treatment with acetazolamide and furosemide does not seem to confer any advantage to the management of posthemorragic hydrocephalus [142].

Whitelaw *et al.* [143] reported a different experience. These authors stated that acetazolamide and furosemide, which both reduce the production of cerebrospinal fluid, have been suggested as non-invasive therapies to reduce hydrocephalus and the need for ventriculo-peritoneal shunting.

Libenson *et al.* [144] evaluated the efficacy of acetazolamide and furosemide in avoiding shunting procedures in preterm infants with posthemorragic hydrocephalus and increased intracranial pressure. Posthemorragic infants were randomized to acetazolamide and furosemide treatment or serial lumbar puncture and monitored until not receiving medications or having undergone shunting. Ten infants with posthemorragic hydrocephalus were randomized to acetazolamide and furosemide treatment and 6 to serial lumbar puncture. Acetazolamide and furosemide therapy is useful in the treatment of preterm infants with posthemorragic hydrocephalus. Because a significant number of infants treated with both acetazolamide and furosemide developed nephrocalcinosis, close monitoring for increased calcium excretion in the urine, or use of acetazolamide without furosemide, is advised.

Eleven premature infants with posthemorragic hydrocephalus were monitored for the development of hypercalciuria during treatment using urine calcium/creatinine ratios [145]. Seven of 11 infants (64%) developed hypercalciuria; five of those seven infants had nephrocalcinosis. Infants who developed nephrocalcinosis had urine calcium/creatinine ratios ranging from 0.5 to 4.0. In all five infants with nephrocalcinosis, renal calculi decreased and urine calcium/creatinine ratios improved after drug therapy was discontinued. The combined use of acetazolamide and furosemide as therapy for posthemorragic hydrocephalus places premature infants at high risk for nephrocalcinosis.

Medical treatment with 100 mg/kg/day acetazolamide and furosemide 1 mg/kg/day can be an effective alternative to shunting by halting progression of hydrocephalus until such time as sutures can become fibrosed and spontaneous arrest can occur [146]. In an approximately selected population older than 2 weeks with hydrocephalus of various origin, the success rate in avoiding shunting is greater than 50%. The dramatic difference between the number of hospitalizations of patients with shunts and those treated medically, and the potential of avoiding shunt dependence would appear to make an initial trial with medical therapy worthwhile.

### 4.14. Side-Effects of Furosemide in Neonates

Water and electrolyte imbalances occur frequently, especially hyponatremia, hypokalemia, and hypochloremic alkalosis after furosemide administration [7]. Hypercalciuria and development of renal calculi occur with long-term furosemide therapy [7]. Furosemide is potentially ototoxic, especially in patients also receiving aminoglycosides [7]. It is important to avoid other ototoxic drugs such as the aminoglycosides in order not to potentiate furosemide ototoxicity [10]. The risk of ototoxicity is dependent on high serum furosemide concentrations, continuous infusion may be used to reduce its incidence [30,147]. In the very preterm infants, t_1/2_ may be as long as 24 h, making progressive drug accumulation possible with repeated use, and this may be a factor in the increased risk of serious late-onset deafness seen in children exposed to sustained treatment in the neonatal period [10]. Premature neonates <32 weeks postmenstrual age have an increased risk of developing high serum furosemide concentrations due to prolonged t_1/2_ and, therefore, dosing schedules should be adjusted for this age group [148]. Furosemide stimulates renal synthesis of prostaglandin E2 [12], thus enhancing, and modifying, renal blood flow. Early use is associated with some increase in the incidence of symptomatic patent ductus arteriosus in infants requiring ventilation for respiratory distress, and this might be due to increased prostaglandin production [10]. Thus, furosemide has an opposite effect to ibuprofen and indomethacin which are the drugs used to close the patent ductus arteriosus. Elective administration of furosemide to any patient with respiratory distress syndrome should be carefully weighed against the risk of precipitation of a symptomatic patent ductus arteriosus. The use of furosemide in infants may lead to nephrocalcinosis and nephrolithiasis, due to high urinary calcium excretion [149].

## 5. Discussion

Furosemide is a loop diuretic because it inhibits activity of the Na^+^-K^+^-2Cl^−^ symporter in the thick ascending limb of the loop of Henle. Other loop diuretics are bumetanide, ethacrynic acid, torsemide, axosemide, piretanide and tripamide [3]. Furosemide is the most used loop diuretic in the neonatal intensive care unit [1,2]. Although the proximal tubule reabsorbs about 65% of filtered Na^+^, diuretics acting only in the proximal tubule have limited capacity because the thick ascending limb has a great reabsorptive capacity and reabsorbs most of the rejectate from proximal tubule. Diuretics acting predominantly at sites past the thick ascending limb also have limited efficacy because only a small percentage of the filtered Na^+^ load reaches these more distal sites. In contrast, inhibitors of Na^+^-K^+^-2Cl^−^ symport in the thick ascending limb are highly efficacious and for this reason are called *high-ceiling diuretics.* The efficacy of inhibitors of Na^+^-K^+^-2Cl^−^ symport in the thick ascending limb of the loop of Henle is due to approximately 25% of the filtered Na^+^ load normally being reabsorbed by the thick ascending limb, and nephron segments past the thick ascending limb do not possess the reabsorptive capacity to rescue the flood of rejectate exiting the thick ascending limb [3].

The extracellular water is 2.4-fold higher in neonates than in adult subjects [17]. In adults, furosemide Vd is 0.13 l/kg [5], suggesting that furosemide is mainly distributed into the extracellular water and in neonates Vd is larger than in adults. In fullterms, Vd of furosemide is larger than in preterms [63,64] suggesting that the fraction of the administered dose of furosemide distributed in periphery is higher in fullterm infants.

In adults, t_1/2_ is 1.3 h [5] and this figure is much less than t_1/2_ of neonates which ranges from 7.7 [61,64] to 26.8 h [63]. Thus, furosemide is eliminated more slowly in neonates than in adults and, consequently, furosemide Cl is lower in neonates. This is a factor that makes progressive drug accumulation possible with repeated use. With the increasing of the postmenstrual age, the elimination of furosemide increases and the tendency of accumulating drug in plasma decreases [6]. The reason for the prolonged half-life of furosemide in newborn infants is the slow renal elimination, related to immature renal function, which is compounded by a reduced metabolic elimination. The kidney maturation drives the furosemide pharmacokinetics.

Drug elimination involves both renal elimination and metabolism. Aranda *et al.* [61] found that furosemide is metabolised into an acidic metabolite and is conjugated with glucuronic acid. In contrast, Tuck *et al.* [62] found that furosemide is not metabolised in neonates. The mid-gestation human fetal liver [56] and kidney [57] have significant levels of glucuronosyl transferase activity and its endogenous substrate adenosine 5’-diphosphoglucuronic acid [150].

In adults, renal elimination of furosemide occurs by glomerular filtration as well as by tubular secretion via a general organic anionic secretory pathway located in the proximal convolute tubule [151,152,153,154]. In neonates, furosemide elimination is decreased because of a low rate of tubular secretion, and in infants with very low body weight, filtration is the major route of renal elimination [6].

It has been suggested giving furosemide by continuous intravenous infusion to pediatric patients following cardiac surgery [82,84,85,88,92]. Appropriate maintenance of patient’s volume status is an important part of the perioperative management of patients undergoing cardiac surgery [79,80,81]. Trials assessing efficacy and safety of continuous *versus* intermittent intravenous infusion of furosemide in pediatric patients after cardiopulmonary bypass surgery revealed that the total furosemide dose administered by continuous intravenous infusion was generally less than the dose by intermittent intravenous administration [82,84,88,92]. The initial rate of the continuous furosemide intravenous infusion is 0.1 mg/kg/h and is preceded by a loading bolus injection of 1 mg/kg in patients with normal renal function and 2 mg/kg in patients with acute renal failure [94].

In the continuous intravenous infusion there is less variability in urine output as well as less losses of sodium and chloride than in the intermittent intravenous infusion of furosemide [82]. The efficacy of furosemide in terms of urinary output is satisfactory in both continuous and intermittent intravenous infusion. However, continuous intravenous infusion of furosemide gives a urinary output per dose of drug significantly larger than after intermittent intravenous infusion with lesser fluctuations and fluid replacement than intermittent infusion [84]. Furosemide administered by continuous infusion is advantageous in the post-operative pediatric patient because of a more predictable urine output with less drug requirement and less urinary loss in sodium and chloride.

When the urine output is <1 mL/kg/h, the dose of furosemide can be increased to 0.4 mg/kg/h [82,84,88,149]. The starting dose for continuous intravenous furosemide infusion ranges from 0.08 to 0.1 mg/kg/h and could be adjusted every 24 h in steps of 0.1 mg/kg/h.

Tolerance to furosemide can be induced through different but complementary homeostatic mechanisms in the kidney [95,96]. The mechanism which underlies the development of tolerance is dehydration. Segar *et al.* [155] stated that tolerance to furosemide appears to be explained by compensatory increased sodium and chloride reabsorption without changes in creatinine clearance. The administration of furosemide enhances diuresis, natriuresis, and chloruresis and overlap the rapid development of tolerance to furosemide in infants with bronchopulmonary dysplasia by blocking the compensatory increase in renal sodium and chloride absorption. The tolerance can be overcome by combination diuretic therapy such as a loop diuretic and a thiazide.

The dosing schedule of continuous intravenous infusion of furosemide in neonates treated with ECMO is largely empirical because of the variable renal function and the altered furosemide pharmacokinetics. van der Vorst *et al.* [9] observed that the dose of the continuous intravenous infusion of furosemide ranged from 0.02 to 0.17 mg/kg/h, and 39% of the patients needed additional diuretic. The evaluated furosemide regimen of 0.2 mg/kg/h preceded by a loading dose of 1 mg/kg is an effective means to obtain rapid and sufficient diuresis without cardiovascular instability in neonates treated with ECMO with a relatively low interindividual variability in urine production.

Some authors found a benefit of inhaled furosemide in preterm infants with respiratory distress syndrome. Other authors found a lack of effect of inhaled furosemide on lung mechanics. Steward *et al.* [11] and Brion and Soll [13] reviewed the risk and benefits of inhaled furosemide in preterm infants with respiratory distress syndrome. These authors concluded that there are no data to support routine administration of furosemide in preterm infants with respiratory distress syndrome. One mg/kg inhaled furosemide does not improve the pulmonary mechanics in ventilated-dependent infants with severe bronchopulmonary dysplasia [102]. Inhaled furosemide has no significant clinical effects in hospitalised infants with viral-bronchitis [103].

Prabhu *et al.* [104] reported different results, these authors stated that a single dose of 1 mg/kg of nebulised furosemide improves pulmonary function in premature infants with evolving chronic lung disease without adverse effects on fluid and electrolyte balance. After aerosolized furosemide (2 mg/kg), static respiratory compliance increased significantly by 24.3% and 23.2% as percentage change from the baseline value at 1 and 2 h (*p* = 0.014 and 0.02, respectively). Also tidal volume increased significantly by 33.8% and 28.7% at 1 and 2 h, respectively (*p* = 0.004 and 0.009; [105]).

Furosemide stimulates renal synthesis of prostaglandin E2 [12], a potent dilator of the ductus arteriosus. After 1 mg/kg furosemide intramuscularly, urinary prostaglandin E2 increased from 17.53 ± 3.37 to 23.73 ± 3.16 ng/12 h (*p* < 0.025). Thus, furosemide has an opposite effect to ibuprofen and indomethacin which are the drugs used to close the patent ductus arteriosus.

Brion and Campbell [109] studied whether furosemide affects the incidence of failure of patent ductal closure after indomethacin administration. These authors observed that there is not enough evidence to support the administration of furosemide to preterm infants treated with indomethacin for symptomatic patent ductus arteriosus. After 1 mg/kg furosemide, prostaglandin E2 excretion rate increased from 0.4 ± 0.04 to 1.3 ± 0.2 ng/mg creatinine. Following the administration of indomethacin to 2 patients with patent ductus arteriosus, the urinary excretion of prostaglandin E2 decreased [111]. These results demonstrate that furosemide enhances urinary excretion of prostaglandin E2 by mechanisms which may reflect an increase in prostaglandin synthesis.

Lee *et al.* [108] administered furosemide (1 mg/kg) to preterm infants with gestational age <34 weeks. Each infant received indomethacin. Furosemide, in combination with indomethacin, increased the incidence of acute renal failure but did not affect the patent ductus closure rate in preterm infants.

Infants who received indomethacin and furosemide had significantly higher urine output (*p* < 0.05) and higher GFR (*p* < 0.05) than those of infants who received indomethacin alone [112]. Furosemide prevented the renal side effects of indomethacin therapy and yet did not affect the efficacy of indomethacin in the closure of the patent ductus arteriosus.

Furosemide may cause ototoxicity in neonates, which may be permanent in some cases. Heidland and Wigand [14] suggested that furosemide should be given at a rate less than 4 mg/min to adult patients to avoid hearing loss. Ototoxicity appears closely related to a prolonged administration and higher total dose of ototoxic drugs, particularly aminoglycosides [124]. Aminoglycosides should not be administered in association with furosemide [10]. Methods of avoiding ototoxicity include slow continuous infusion rather than bolus injection, use of divided oral dose regimens, and the measurement of blood levels to avoid exceeding 50 µg/mL furosemide in neonates [125]. If a diuretic response cannot be obtained using the above measures, the substitution of another diuretic such as bumetanide is suggested to maintain therapeutic response and minimize the ototoxicity [125].

Preterm infants treated with chronic furosemide may develop intra-renal calcification [134]. The pathogenesis of nephrocalcinosis in very low birthweight infants may depend on the underdevelopment of renal function. Renal injury in early life may lead to hypertension and renal disease in adulthood [127]. Nephrocalcinosis in the preterm infants was associated with impaired renal tubular function and a shorter kidney length in the first year of life [127]. The risk of nephrocalcinosis is 1.65-fold higher per 100 g lower weight and 4.5-fold higher per mmol/l of urinary calcium concentration [129].

Nasseri *et al.* [128] using logistic regression analysis, observed that family history of renal stone and urine calcium/creatinine ratio are the major risk factors of nephrocalcinosis in very low birthweight neonates. Factors associated with nephrocalcinosis include severity of respiratory illness, patent ductus arteriosus, oxygen dependency and furosemide therapy [130]. Furosemide-related renal calcifications in very low birthweight infants may lead to glomerular and tubular dysfunction [133]. Discontinuity of furosemide therapy is associated with resolution of renal calcifications. Furosemide therapy is responsible for cases of iatrogenic nephrocalcinosis [135] and renal calcification has rates of calcium excretion 10 to 20 times the normal [137]. Furosemide or dexamethasone therapy is associated with renal calcification [138] and extremely premature sick infants requiring long-term ventilation are more likely to have calcification. Furosemide administered in association with acetazolamide is neither effective nor safe in treating posthemorragic hydrocephalus.

## 6. Conclusions

Kidney maturation drives furosemide pharmacokinetics. There is a remarkable interindividual variability in the kinetic parameters of furosemide in neonates. Furosemide t_1/2_ is longer and Cl is smaller in preterm than fullterm infants. t_1/2_ of furosemide shortens and Cl increases with postnatal development. Continuous intravenous infusion of furosemide in pediatric patients undergoing cardiac surgery is preferable to intermittent intravenous infusion. The continuous infusion of furosemide in ECMO is required to warrant urine production. Furosemide may be administered by inhalation; nebulised furosemide may improve pulmonary function in premature infants. However, some authors found no effect of inhaled furosemide on lung mechanics. Furosemide stimulates the synthesis of prostaglandin E2 and the administration of furosemide to preterm infants may yield a patent ductus arteriosus. Infants with low birthweight treated with chronic furosemide are at risk for the development of intra-renal calcification. Implementation of calcium-free hemodialysis resulted in a rapid reduction of serum calcium concentration. Some authors observed that acetazolamide and furosemide confer some advantages to the posthemorragic treatment of the hydrocephalus. Other authors found a lack of effect of these drugs on the posthemorragic hydrocephalus. The effect of acetazolamide and furosemide on the posthemorragic hydrocephalus is still uncertain.
